# Incidence of wound dehiscence after keratoplasty: a meta-analysis of observational studies

**DOI:** 10.3389/fmed.2023.1187555

**Published:** 2023-08-30

**Authors:** Na Zheng, Wenjing He, Siquan Zhu

**Affiliations:** ^1^Eye School, Chengdu University of Traditional Chinese Medicine, Chengdu, China; ^2^Big Data Research Center, University of Electronic Science and Technology, Chengdu, China; ^3^Department of Ophthalmology, Beijing Anzhen Hospital, Capital Medical University, Beijing, China

**Keywords:** wound dehiscence, penetrating keratoplasty, deep anterior lamellar keratoplasty, visual acuity, meta-analysis

## Abstract

**Background:**

The comprehensive investigation of the association between keratoplasty and wound dehiscence remains limited, despite corneal disease being a leading cause of visual impairment.

**Methods:**

A meticulous search strategy was executed across prominent databases such as Web of Science, PubMed, Cochrane Library, and Embase. Data relevant to our research objective were extracted from eligible studies. The methodological quality of each study was assessed using the ROBINS-I tool, while statistical analysis was conducted utilizing STATA 17.0. To evaluate potential publication bias, the Funnel plot and Egger’s test were employed.

**Results:**

A total of 11 articles were deemed suitable for inclusion in our analysis. Our findings indicate that the overall incidence of wound dehiscence following keratoplasty was estimated to be 1.9% (95% CI: 0.013, 0.026), although substantial heterogeneity was observed (*I*^2^ = 72.798%). Notably, developed countries exhibited a higher incidence of wound dehiscence compared to their developing counterparts. Furthermore, the occurrence of wound dehiscence was found to be lower in deep anterior lamellar keratoplasty (DALK) procedures when compared to penetrating keratoplasty (PK). Analysis utilizing Egger’s linear regression method yielded no evidence of publication bias (*p* = 0.91). Moreover, within the first year post-keratoplasty, approximately 31.4% of patients experienced wound dehiscence (95% CI: 0.149, 0.503), and 43.1% exhibited a decline in best-corrected visual acuity (BCVA) (95% CI, 0.341, 0.522).

**Conclusion:**

The results of our study unveiled the occurrence rate of wound dehiscence following keratoplasty, exhibiting variations based on economic level and the specific surgical procedure employed. Furthermore, onset time of wound dehiscence and visual acuity warrant consideration.

## Introduction

Corneal disease ranks as the second leading cause of blindness on a global scale. Patients diagnosed with severe corneal disease often necessitate keratoplasty, a surgical procedure involving the replacement of damaged corneal tissue with a healthy, functional cornea. This restorative intervention aims to enhance vision and manage corneal disease in the affected eye (1, 2). Postoperative complications that may arise following keratoplasty encompass graft failure, rejection or dislocation, suture-related issues, infectious keratitis, glaucoma, cataract, refractive errors, and more. Among these, wound dehiscence (WD) has garnered considerable attention due to the vulnerability of postoperative wound tissue to external forces, which can potentially result in significant ocular structural damage or even extreme outcomes such as eyeball atrophy (3–6).

Keratoplasty is broadly categorized into two types: full-thickness (penetrating) and partial-thickness (lamellar). Penetrating keratoplasty (PK) involves the complete excision of the diseased cornea, which is then substituted with a donor cornea of the same size or slightly larger. The donor cornea is meticulously sutured to the recipient cornea using nylon thread. On the other hand, deep anterior lamellar keratoplasty (DALK) exclusively removes the surface tissue of the diseased cornea, leaving the deep, intact cornea of the recipient as the transplant bed. The donor cornea is then skillfully sewn onto the recipient’s cornea, ensuring size and thickness congruence. Since DALK does not penetrate the anterior chamber, it is generally regarded as a procedure with fewer complications, minimizing interference with intraocular tissue (7–14).

Although the incidence of WD following keratoplasty has been reported in various geographical regions worldwide, the findings have shown considerable variation. Consequently, the objective of this study is to estimate the overall incidence of wound dehiscence after both PK and DALK procedures. Additionally, we aim to examine the onset time of WD and the impact of keratoplasty on visual acuity. Furthermore, we endeavor to assess the incidence variations based on the type of surgical approach and the economic level of the populations under investigation.

## Materials and methods

### Selection criteria

This study adhered to the established guidelines presented in the Cochrane Handbook for Systematic Reviews of Interventions. Eligible studies were required to fulfill the following criteria: (1) report the total number of wound dehiscence cases following PK or DALK procedures; (2) provide comprehensive information on the patients’ underlying diseases, cause of trauma, visual acuity, and other relevant characteristics; (3) demonstrate no significant differences in the baseline characteristics of the included patients; (4) have been completed by the end of 2022, with a complete dataset available; and (5) adhere to the standard time criteria. Studies that failed to meet these criteria were excluded from the analysis.

### Search methods

Language restrictions were not applied to the included studies, and both published and unpublished studies were deemed eligible. A systematic search was conducted across electronic databases, including Web of Science, PubMed, Cochrane Library, and Embase, encompassing their inception until December 2022. The search strategy employed a combination of relevant keywords, such as “wound dehiscence,” “graft dehiscence,” “keratoplasty,” “penetrating keratoplasty,” and “deep anterior lamellar keratoplasty.” Two reviewers independently screened titles and abstracts for eligibility, followed by a thorough examination of full-text articles for potentially eligible studies. Any disagreements between the reviewers were resolved through consensus.

### Data extraction and risk of bias

Two independent reviewers employed standardized data collection forms to extract relevant data from eligible studies, encompassing study design, patient characteristics, surgical procedures, primary diseases, and duration of follow-up. The primary outcome of interest was the incidence of wound dehiscence after keratoplasty, while secondary outcomes included the occurrence of decreased best-corrected visual acuity (BCVA) measured in terms of Snellen or LogMAR units, as well as the occurrence of wound dehiscence within 1 year post-surgery. The risk of bias in observational studies was evaluated using the Risk of Bias in Non-randomized Studies of Interventions (ROBINS-I) tool (15). Both reviewers independently conducted the risk of bias assessments, resolving any discrepancies through consensus. In cases where a consensus could not be reached, a third author was consulted.

### Data synthesis and analysis

The statistical analysis for this study was performed using STATA 17.0 (STATA Corporation LP). A significance level of *p* < 0.05 was considered for all analyses, unless otherwise specified. Hedges’ effect size (ES) and corresponding 95% confidence intervals (CI) were calculated. If the data was dichotomized, the odds ratio for improvement was extracted or calculated and converted into an effect size. Random effects meta-analysis was utilized to combine study estimates, with weights determined using the inverse variance method. Heterogeneity was assessed using the I2 statistic, and the *p*-value was calculated using the Q test. Publication bias was evaluated using funnel plots, and Egger’s test was employed to detect any potential asymmetry in the plots.

## Results

### Study characteristics

**Figure 1:**
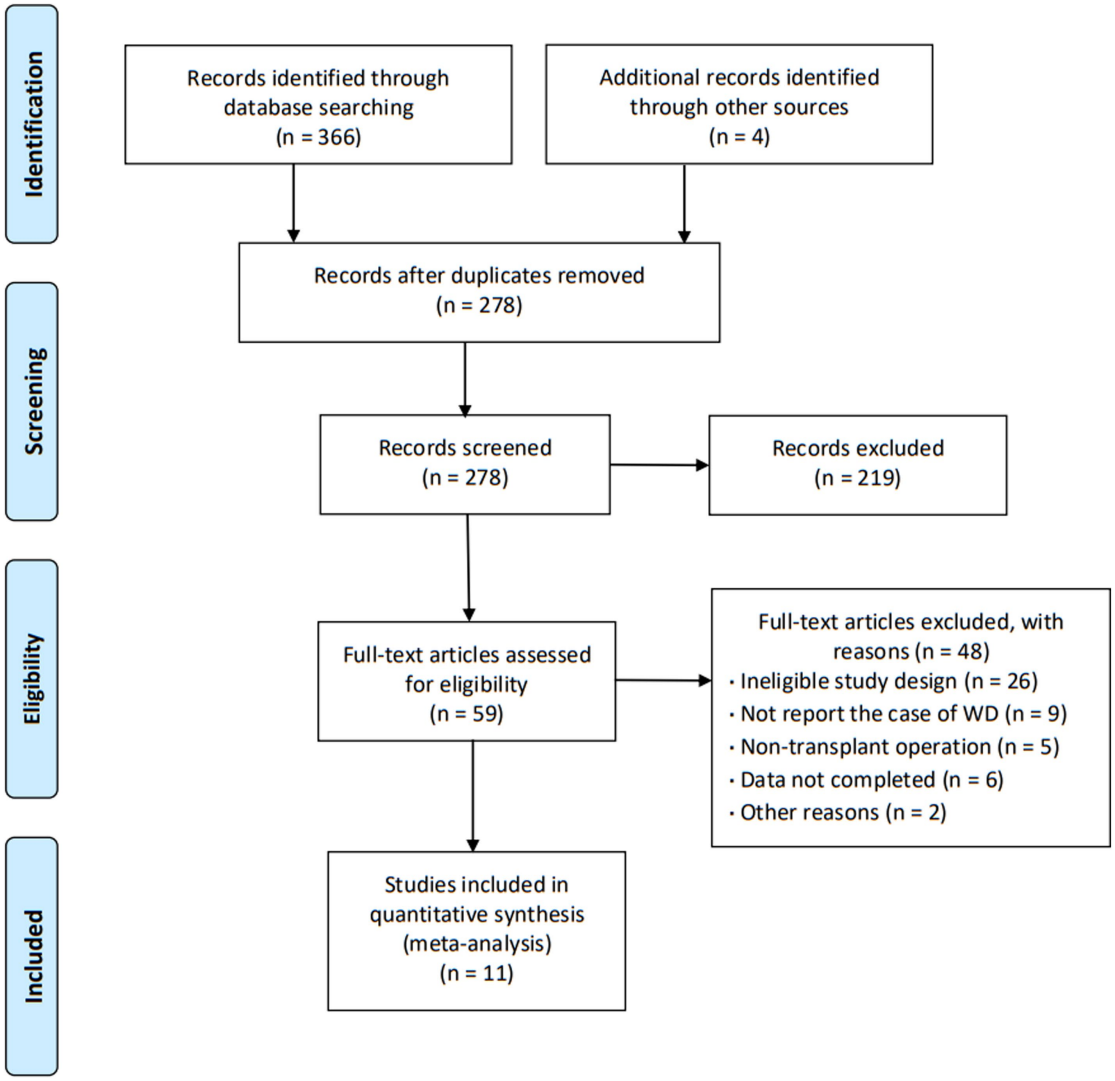
Flow diagram.

The initial search yielded a total of 370 articles. After screening the abstracts and titles, 59 articles underwent full-text review, resulting in the inclusion of 11 articles (16–26) that encompassed a collective sample size of 7,878 patients (Figure 1). These 11 articles provided comprehensive insights into the characteristics of patients experiencing wound dehiscence after keratoplasty. The majority of the included articles (90.9%) were retrospective observational studies, and the agreement between reviewers regarding eligibility was deemed satisfactory. The publication dates of the included studies ranged from 2002 to 2020, with three articles specifically addressing DALK. The specific characteristics of the included articles are outlined in Table 1.

**Table 1:** Study characteristic.

Study	Country	Type of study	Study population (wound dehiscence)				Type of keratoplasty	Follow-up time
			WD patients (total)	Age (year)	Gender (male/female)	Original indications		
Meyer and McGhee (16)	New Zealand	Retrospective	30 (1,294)	PK: 46.0 ± 20.2 DALK: 35.9 ± 15.4	NR	Keratoconus (54.3%), scarring (13.6%), bullous keratopathy (11.3%), corneal dystrophy (8.4%), ulceration/keratitis (7.8%) and others (3.0%)	Penetrating keratoplasty or deep anterior lamellar keratoplasty	PK: 7.5 ± 4.1 years DALK: 5.8 ± 4.0 years
Wang et al. (17)	China	Retrospective	31 (3,017)	44.6 ± 18.3	26/4	Keratoconus (50.0%), fungal keratitis (25.0%), interstitial keratitis (12.5%), and ocular chemical injury (12.5%)	Penetrating keratoplasty or lamellar keratoplasty	PK: 45.0 ± 36.4 months LK: 48.4 ± 66.2 months
Ma et al. (18)	USA	Retrospective	30	56 ± 22	20/10	Keratoconus (35%), corneal scarring (16%) and corneal scarring (12%)	Penetrating keratoplasty	0.8 years (0.02–2.67 years)
Bamashmus et al. (19)	Yemen	Retrospective	53	4 ~ 65	41/12	Keratoconus (75.5%), aphakic bullous keratopathy (7.5%), corneal dystrophy (5.7%), traumatic corneal opacity (5.7%), perforated corneal ulcer (3.8%), and herpetic corneal opacity (1.8%)	Penetrating keratoplasty	NR
Das et al. (20)	Australia	Retrospective	19 (624)	65 ± 15	13/6	The most indication for the original graft was keratoconus	Penetrating keratoplasty	NR
Renucci et al. (21)	USA	Retrospective	51	69.5 (10 ~ 98)	NR	Bullous keratopathy (51%), infectious keratitis (11%), Fuchs dystrophy (9%), and keratoconus (9%)	Penetrating keratoplasty	NR
Foroutan et al. (22)	Iran	Retrospective	7 (490)	20.6 (10 ~ 30)	6/1	Keratoconus (42.86%), macular dystrophy (28.57%), and corneal scarring (28.57%)	Penetrating keratoplasty	NR
Kartal et al. (23)	Turkey	Retrospective	26 (1,625)	40.7 ± 19.6	14/12	The most frequent primary PK indication was corneal scar	Penetrating keratoplasty	6–117 months
Sari et al. (24)	Turkey	Retrospective	11 (338)	30.6 ± 5.4	7/4	Keratoconus (54.5%), stromal dystrophies (27.3%), and herpetic keratitis (18.1%)	Deep anterior lamellar keratoplasty	6.09 ± 2.77 years
Abou-Jaoude et al. (25)	USA	Retrospective	5 (324)	68.4 (56 ~ 80)	1/4	Keratoconus (40%), pseudophakia bullous keratopathy (60%)	Penetrating keratoplasty	24.5 ± 15 months
Huang et al. (26)	China	Retrospective	32	37 ± 16	31/1	The most indication for the original graft was keratoconus and herpes simplex virus keratitis	Penetrating keratoplasty	8.5 ± 3.4 months

WD, Wound dehiscence; PK, Penetrating keratoplasty; LK, Lamellar keratoplasty; DALK, Deep anterior lamellar keratoplasty; NR, Not report.

### Wound dehiscence

**Figure 2:**
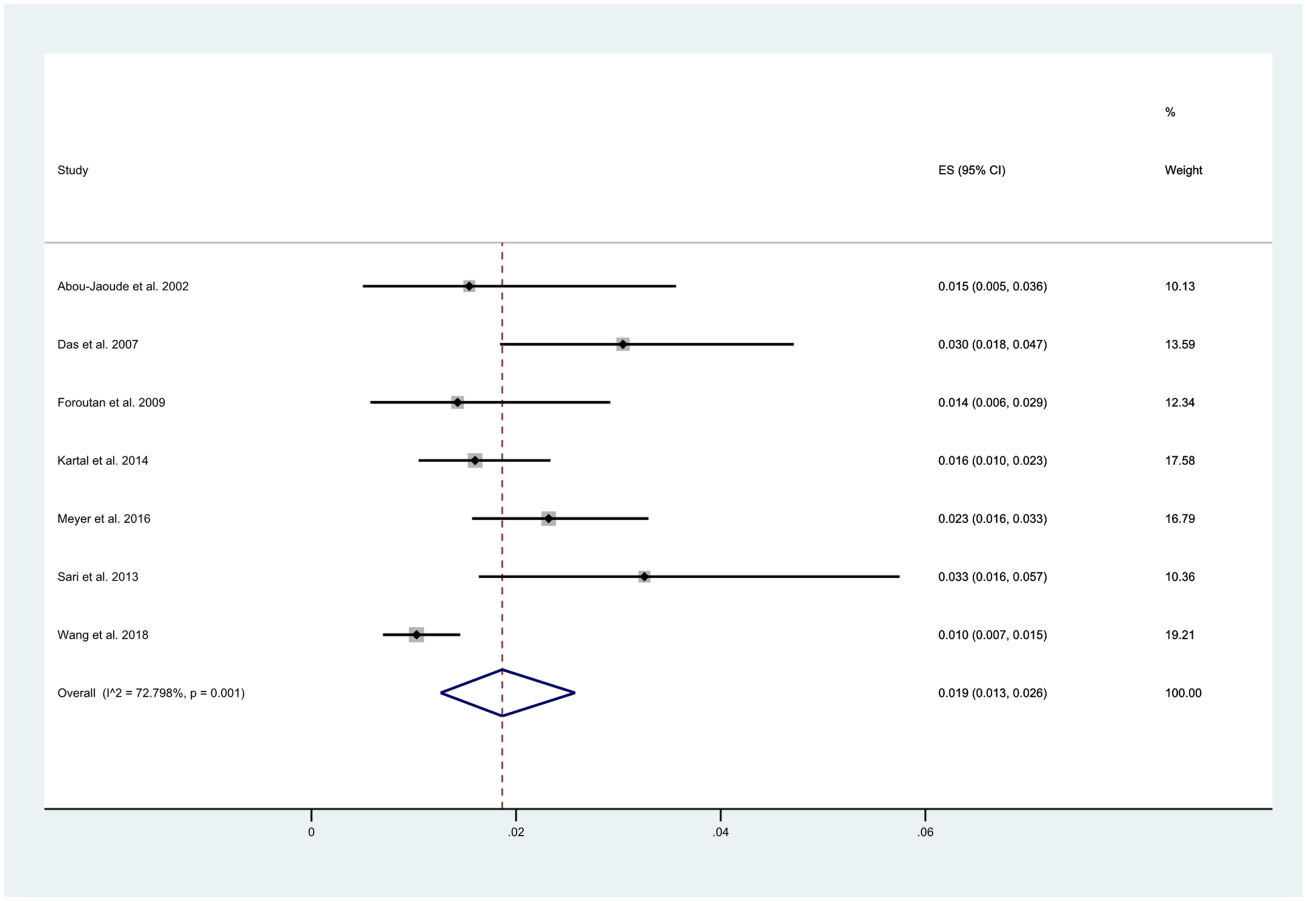
Incidence of wound dehiscence after keratoplasty.

O Among the 11 reviewed articles, seven reported the total number of patients experiencing wound dehiscence. The overall incidence of wound dehiscence was estimated to be 1.9% (95% CI: 0.03, 0.26), as depicted in Figure 2. However, this estimate displayed a relatively high level of heterogeneity (*I*^2^ = 72.8%).

### Wound dehiscence in developing vs. developed countries

**Figure 3:**
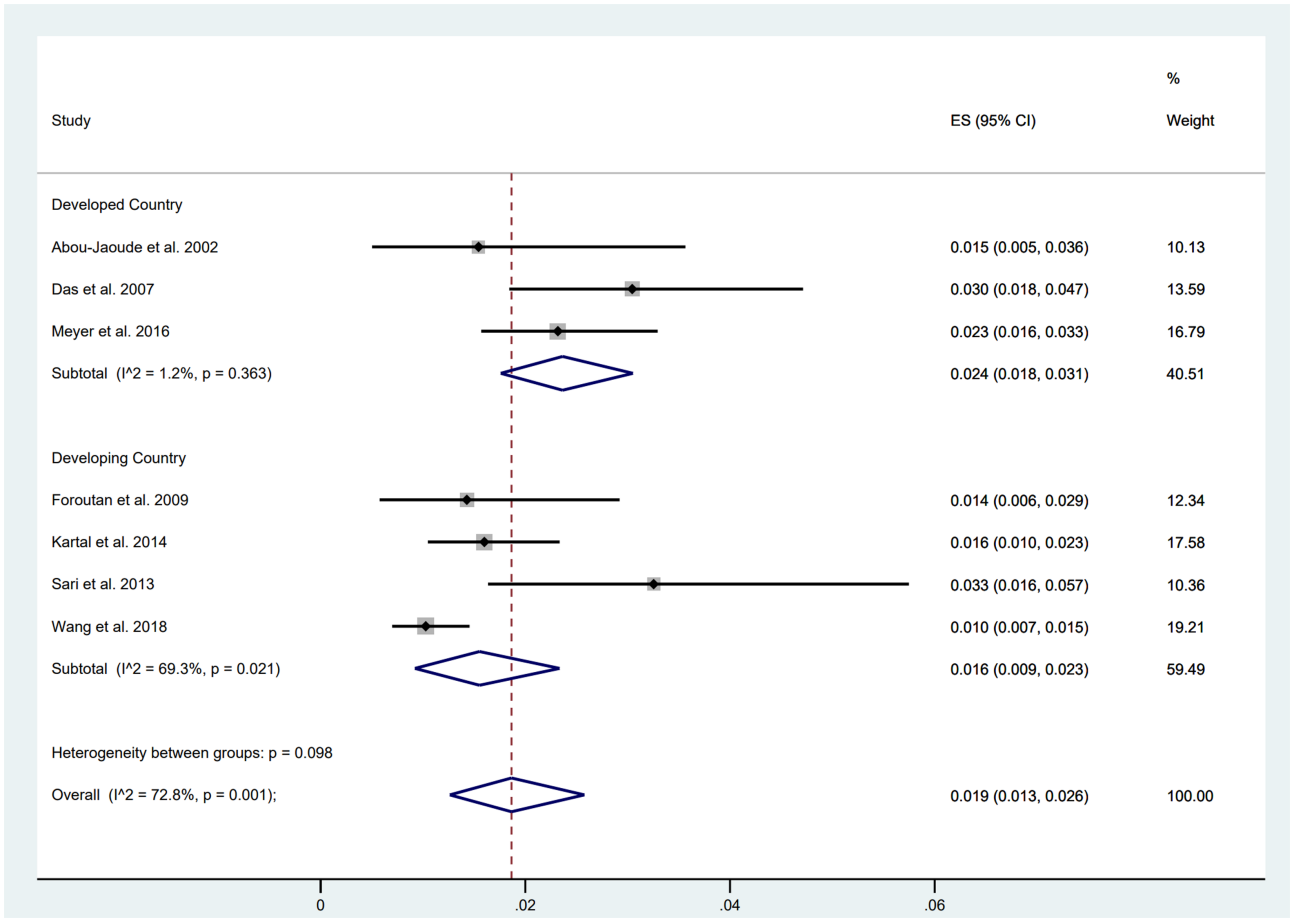
Incidence of WD in developing and developed countries.

Subgroup analyses based on the economic level of countries were conducted, comprising five studies from developed countries (including the United States, Australia, and New Zealand) and six studies from developing countries (including China, Turkey, and Iran). The results indicated a higher incidence of wound dehiscence in developed countries (ES = 0.024, 95% CI: 0.018, 0.031) compared to developing countries (ES = 0.016, 95% CI: 0.009, 0.023) (Figure 3).

### Wound dehiscence after PK vs. DALK

**Figure 4:**
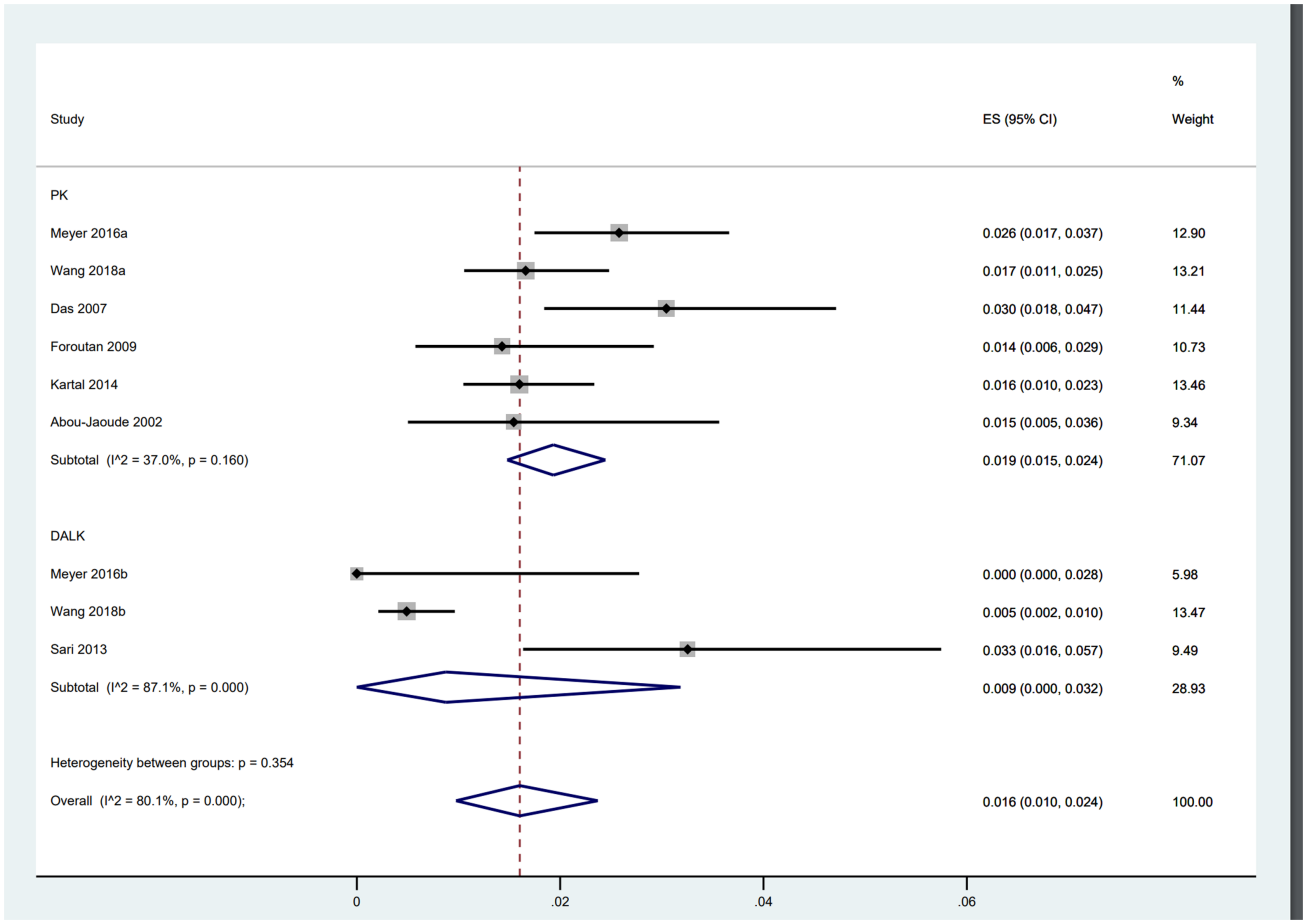
Incidence of WD after PK and DALK.

Of the included studies, six focused on PK involving 5,611 patients, while three trials examined DALK with a total of 2,101 patients. The analysis revealed that patients undergoing PK (ES = 0.019, 95% CI: 0.015, 0.024) were more than twice as likely to experience wound dehiscence compared to those undergoing DALK (ES = 0.009, 95% CI: 0.000, 0.032) (Figure 4).

### Onset time of wound dehiscence

**Figure 5:**
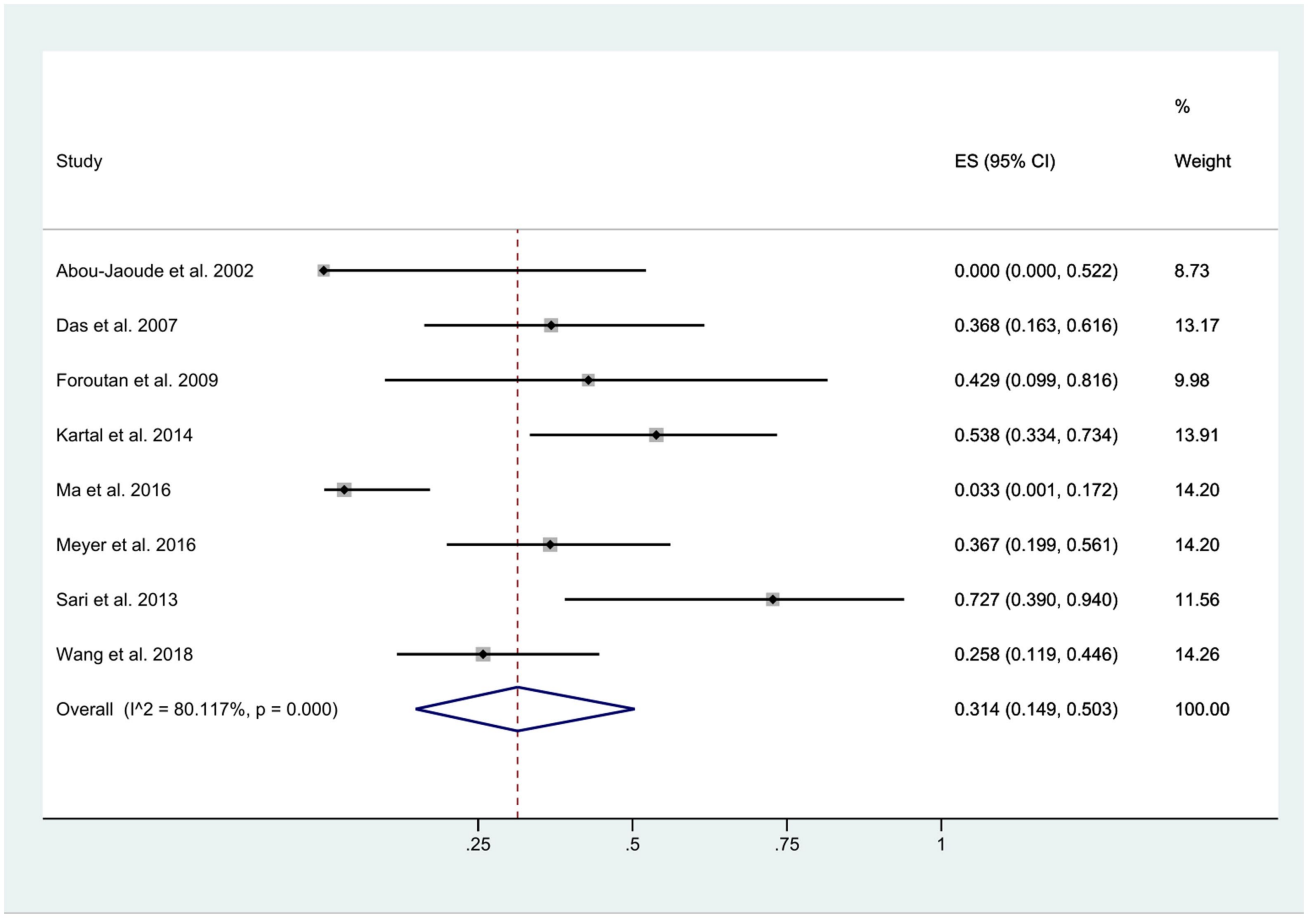
Occurrence of WD less than 1 year after keratoplasty.

The time interval between the initial surgery and the occurrence of wound dehiscence exhibited considerable variability, ranging from 1 week to 16 years. Regardless of whether wound dehiscence was spontaneous, caused by a fall, or a result of external impact, it was documented after keratoplasty. Within 1 year post-surgery, approximately 31% of patients experienced wound dehiscence (95% CI: 0.15, 0.50). However, this estimate displayed a high level of heterogeneity (*I*^2^ = 80.12%), as illustrated in Figure 5.

### Visual acuity

**Figure 6:**
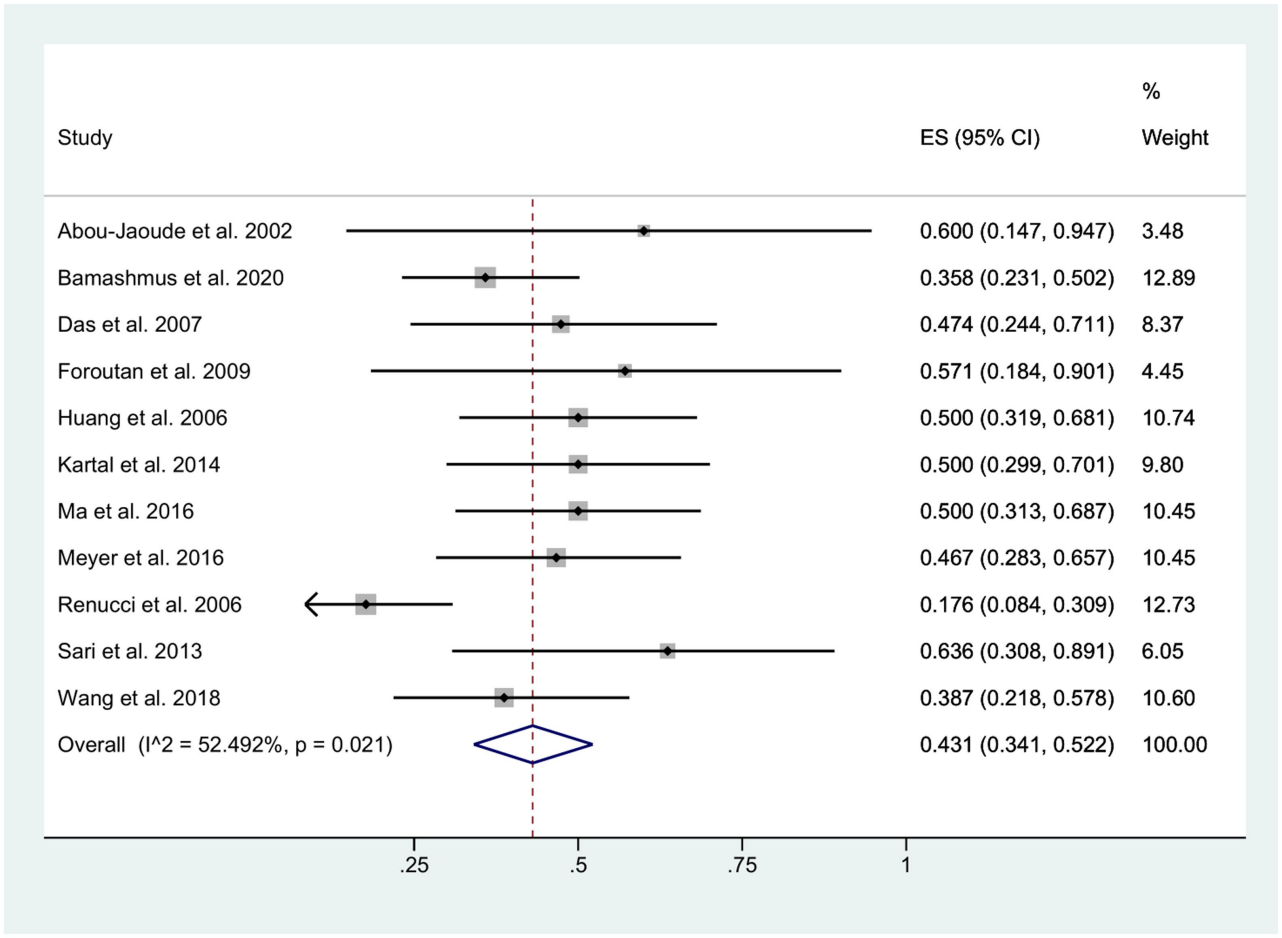
Decreased BCVA.

The final visual outcome was determined based on measurements obtained at the patients’ last clinical visit. Among the eyes with available pre- and post-fissure data, the mean BCVA was 20/400 for patients with retained lenses and 20/800 for patients with aphakia. Following the occurrence of wound dehiscence, approximately 43% of patients exhibited a decrease in BCVA (95% CI: 0.34, 0.52; *I*^2^ = 52.49%), as depicted in Figure 6. Moreover, in the DALK group, 57.9% (11/19) of the patients exhibited a decline in BCVA, whereas in the PK group, this proportion was 39.9% (110/276).

### Risk of bias and publication bias

**Figure 7:**
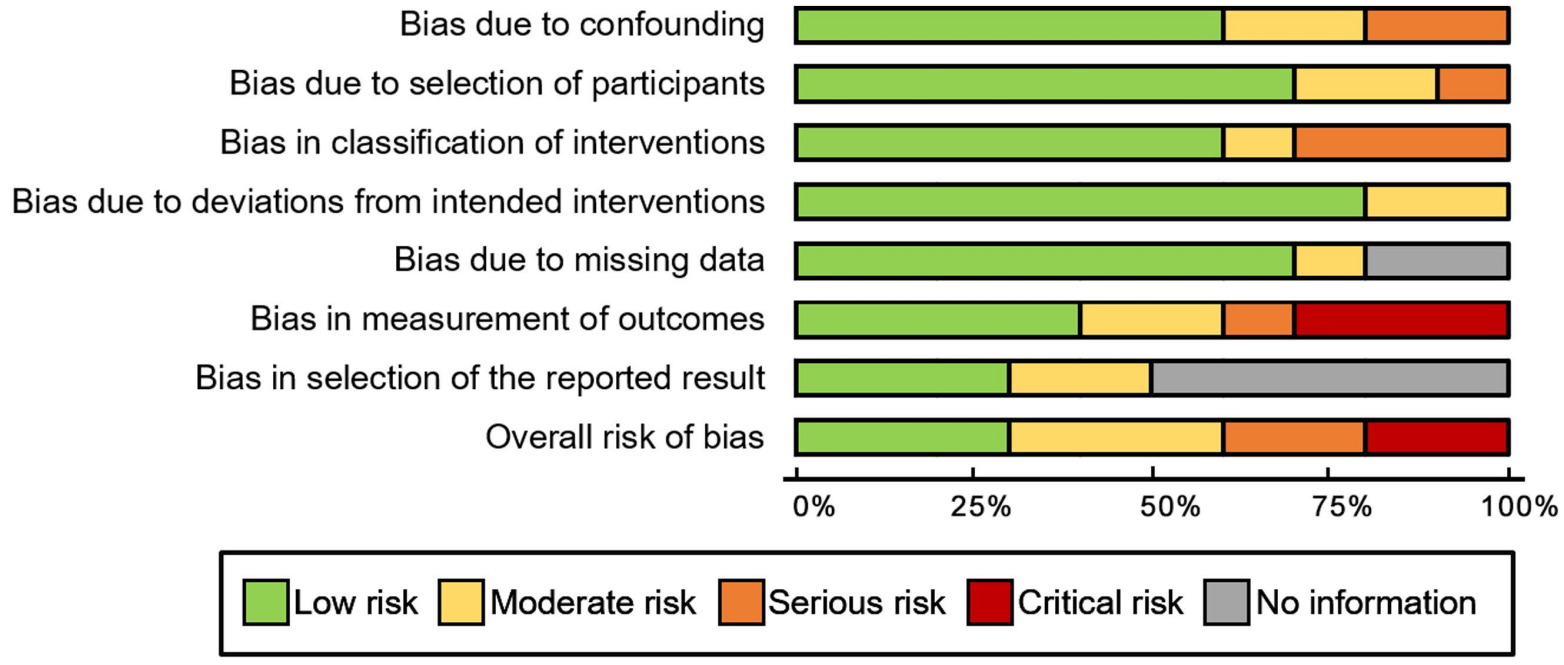
Risk of bias analysis (ROBINS-I).

**Figure 8:**
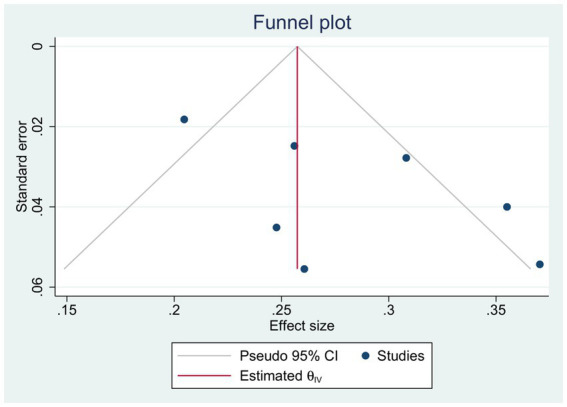
Funnel plot.

For observational studies, the risk of bias was assessed utilizing the ROBINS-I tool. One study by Huang et al. (26) exhibited a critical risk of bias due to concerns related to confounding factors, intervention classifications, and potential deviations from the assigned intervention. Further details regarding the results of the risk of bias analysis are presented in Figure 7. There was no evidence of publication bias for the primary endpoint of adverse events, as indicated by Egger’s linear regression method (*p* = 0.91). Additionally, a funnel plot was constructed to evaluate the incidence of wound dehiscence (Figure 8).

## Discussion

This study aimed to provide an estimation of the incidence of wound dehiscence following keratoplasty, with a specific focus on comparing the incidence between developed and developing countries, as well as between PK and DALK. Furthermore, the study examined additional outcomes associated with keratoplasty, such as the occurrence of wound dehiscence within 1 year post-surgery and the impact on patients’ BCVA.

Keratoplasty, despite its potential therapeutic benefits, warrants heightened attention due to the presence of significant postoperative complications, such as WD. While Descemet’s membrane in the eyes possesses remarkable elasticity and resistance to trauma, the adhesion between Descemet’s membrane and the stroma becomes considerably delicate following ophthalmic surgeries, rendering it susceptible to WD when subjected to external forces (27). WD predominantly manifests at the graft-host junction, where tissue stability and stretching capacity are considerably compromised, not only in comparison to the normal cornea but also when contrasted with the corneal scar formed during the healing process following corneal laceration (28, 29). WD represents a deleterious complication associated with weak graft-host connections, as well as various contributory factors including inappropriate wound anastomosis, insufficient interfacial blood supply, prolonged topical steroid therapy, and suture-related complications (30).

Indications for keratoplasty exhibit significant variation across different countries and regions. In Western developed countries, bullous keratopathy, keratoconus, and Fuchs endothelial dystrophy rank as the most prevalent indications (31–35). Within the United States, the primary indications encompass bullous keratopathy, secondary keratoplasty, keratoconus resulting from various factors, and keratoconus (36). In New Zealand, the leading indications comprise keratoconus, bullous keratopathy, secondary transplantation, viral keratitis, and trauma, whereas in Germany, the most common indications include keratoconus, keratoplasty associated with diverse factors, and bullous keratopathy (37). Infectious keratopathy prevails in Southeast Asia and India, with Taiwan reporting leukoplakia, infectious corneal disease, and bullous keratopathy as the top three reported cases, consistent with the order of reported cases in India (38).

Presently, DALK stands as the recommended procedure, particularly for young and active patients afflicted with keratoconus and corneal scar involvement within the stroma. In this approach, the host Descemet’s membrane remains undisturbed, offering potential advantages in terms of postoperative healing time and reduced risk of wound dehiscence (39, 40). The presence of an intact Descemet’s membrane serves as a protective physical barrier against intraocular tissue damage, thus either reinforcing the resistance to traumatic dehiscence or mitigating the severity of ocular injury in such events. However, it is important to acknowledge that relative weakness at the graft-host junction might still persist after DALK, and Descemet’s membrane could be susceptible to wrinkling post-surgery, potentially influencing the incidence of postoperative complications, including wound dehiscence (41, 42). Notably, research by Sari et al. (24) highlights that only 3 out of 11 eyes exhibited an intact Descemet’s membrane following DALK. Despite previous studies demonstrating the efficacy of keratoplasty in enhancing BCVA and achieving favorable refractive outcomes, there were no significant differences observed between DALK and PK in this regard (43, 44).

The primary mechanism underlying the unfavorable prognosis following wound dehiscence is the subsequent structural alterations it induces in the anterior and posterior segments of the eyeball, including elevated intraocular pressure, vitreous hemorrhage, and complications such as choroid and retinal detachment. Consequently, the prevention of wound dehiscence following transplantation assumes paramount importance over its treatment. It is crucial to protect the surgical eye from injury throughout one’s lifetime after keratoplasty. Although achieving satisfactory visual outcomes is uncommon, there is still hope for successful outcomes as long as the integrity of the posterior segment structure is preserved and no further complications arise. Clinical ophthalmologists should maintain a constant awareness of the potential causes of unusual wound dehiscence and the associated complications following keratoplasty. The significance of wound dehiscence after corneal transplantation should be emphasized during preoperative consultations, particularly among young and active male patients. As part of the treatment approach, patients should be fully informed about the risks associated with wound dehiscence and its potential serious consequences. Comprehensive patient education and awareness programs should be implemented, underscoring the importance of wearing protective glasses. Appropriate activity restrictions should be advised after surgery, especially for high-risk groups such as athletes, manual workers, and children, while the use of wound healing-inhibiting medications like corticosteroids should be minimized in patients with corneal bed tissue edema (45, 46).

This systematic review is subject to several limitations that should be acknowledged. Firstly, the majority of the included studies were retrospective in nature, and some exhibited lower methodological quality. This may introduce potential biases and limit the strength of the conclusions drawn. Additionally, the exclusion of data from patients lost to follow-up may introduce a selection bias, as only data from patients who completed the follow-up period were included, potentially impacting the comparison of the two surgical methods. Secondly, the presence of small sample sizes in the excluded studies is another limitation of this analysis. Smaller studies inherently carry a higher risk of random sampling error, which can influence the overall results and introduce uncertainty. Thirdly, it is important to note that in one of the included studies, the distribution of the number of patients and follow-up time between the PK and DALK groups was not even. This uneven distribution may introduce a potential confounding factor that could influence the interpretation of the findings.

## Conclusion

In the existing body of research exploring various aspects of keratoplasty surgery, including complications, types of trauma, age and sex distribution, and visual prognosis, a comprehensive investigation into the incidence and outcome of traumatic and spontaneous wound dehiscence following keratoplasty remains incomplete. Our study aimed to address this knowledge gap by examining the occurrence rate of wound dehiscence after keratoplasty, taking into account factors such as economic level and the specific surgical procedure employed. Moreover, we assessed the onset time of wound dehiscence and the impact of keratoplasty on visual acuity. Considering that corneal transplantation disrupts the natural structural integrity of the cornea, it is crucial to educate patients on the measures necessary to safeguard the graft from potential trauma.

## Author contributions

SZ designed the study. NZ and WH collected and analyzed the data. NZ drafted the manuscript. SZ and WH revised the manuscript. All authors contributed to the article and approved the submitted version.

## Conflict of interest

The authors declare that the research was conducted in the absence of any commercial or financial relationships that could be construed as a potential conflict of interest.

## Publisher’s note

All claims expressed in this article are solely those of the authors and do not necessarily represent those of their affiliated organizations, or those of the publisher, the editors and the reviewers. Any product that may be evaluated in this article, or claim that may be made by its manufacturer, is not guaranteed or endorsed by the publisher.
